# Overexpression of preeclampsia induced microRNA-26a-5p leads to proteinuria in zebrafish

**DOI:** 10.1038/s41598-018-22070-w

**Published:** 2018-02-26

**Authors:** Janina Müller-Deile, Patricia Schröder, Lynne Beverly-Staggs, Rebecca Hiss, Jan Fiedler, Jenny Nyström, Thomas Thum, Hermann Haller, Mario Schiffer

**Affiliations:** 1 Department of Medicine/Nephrology, Hannover Medical School, Hannover, Germany; 2 Mount Desert Island Biological Laboratory, Salisbury Cove, Maine, USA; 3 Institute of Molecular and Translational Therapeutic Strategies, Hannover Medical School, Hannover, Germany; 4 Department of Physiology, Institute of Neuroscience and Physiology, the Sahlgrenska Academy, University of Gothenburg, Gothenburg, Sweden

## Abstract

So far the pathomechanism of preeclampsia in pregnancy is focussed on increased circulating levels of soluble fms-like tyrosin kinase-1 (sFLT-1) that neutralizes glomerular VEGF-A expression and prevents its signaling at the glomerular endothelium. As a result of changed glomerular VEGF-A levels endotheliosis and podocyte foot process effacement are typical morphological features of preeclampsia. Recently, microRNA-26a-5p (miR-26a-5p) was described to be also upregulated in the preeclamptic placenta. We found that miR-26a-5p targets VEGF-A expression by means of PIK3C2α in cultured human podocytes and that miR-26a-5p overexpression in zebrafish causes proteinuria, edema, glomerular endotheliosis and podocyte foot process effacement. Interestingly, recombinant zebrafish Vegf-Aa protein could rescue glomerular changes induced by miR-26a-5p. In a small pilot study, preeclamptic patients with podocyte damage identified by podocyturia, expressed significantly more urinary miR-26a-5p compared to healthy controls. Thus, functional and ultrastructural glomerular changes after miR-26a-5p overexpression can resemble the findings seen in preeclampsia and indicate a potential pathophysiological role of miR-26a-5p in addition to sFLT-1 in this disease.

## Introduction

Preeclampsia is a multi-systemic disease in pregnancy that significantly contributes to maternal and fetal morbidity and mortality. Renal involvement in preeclampsia includes proteinuria, podocyturia, elevated blood pressure, edema, glomerular capillary endotheliosis, and thrombotic microangiopathy^1^.

One of the major causes in the pathophysiology of preeclampsia is an excess level of circulating soluble fms-like tyrosin kinase-1 (sFLT-1) produced by the placenta that binds circulating vascular endothelial growth factor A (VEGF-A)^2–6^. Glomerular VEGF-A is predominately produced by podocytes^7^ and glomerular endothelial cells are dependent on VEGF-A to keep their proper phenotype and function. Podocytic VEGF-A binds to its receptors on glomerular endothelial cells by diffusive flux against the flow of glomerular filtration^7^ and also acts on podocytes in an autocrine manner^8^. Alterations in glomerular VEGF expression result in endothelial as well as in podocyte damage, thus a tightly orchestrated expression of glomerular VEGF is critical for maintaining normal glomerular structure and integrity^8–10^.

Similarly, the depletion of VEGF-A by anti-VEGF-therapy leads to features of thrombotic microangiopathy with swollen endothelial cells and abnormal podocyte morphology^10–12^. Patients under anti-VEGF-therapy can present with proteinuria, podocyturia, elevated blood pressure and edema which resembles signs and symptoms typically seen in preeclampsia. Furthermore, sFlt-1 overexpression that antagonizes Vegf-A caused symptoms of preeclampsia in an animal model^13^. However, sFlt-1 levels in this animal model were two orders of magnitude higher compared to serum levels detected in women with preeclampsia. Recently micro-RNAs (miRs) were found to play an important role in gene regulation and therefore seem to be promising candidates involved in glomerular diseases. MiRs are non-coding molecules with a length of 21 to 23 nucleotides. They act by binding to the 3′ untranslated region (3′ UTR) of target messenger RNAs and thereby inhibit their translation^14^. Because of their small size miRs can cross blood–brain, placental and glomerular filtration barrier and appear in different body fluids^15^.

We hypothesize that glomerular damage in preeclampsia could be caused by miRs upregulated in this disease, in addition to circulating sFLT-1 levels. Preeclampsia related miRs have been described in serum and placenta tissue previously^16–18^.

*Choi et al*., described several miRs to be dysregulated in placentas of preeclamptic patients that seemed to be closely associated with the early pathogenesis of preeclampsia^19^. Higher levels of miR-26a-5p were previously detected in placentas and plasma from preeclamptic women^20^. Chai and co-workers detected that expression of VEGF-A was inversely correlated with miR-26a-5p expression in hepatocellular carcinoma and that miR-26a-5p modulated angiogenesis of hepatocellular carcinoma through the PIK3C2α/Akt/HIF-1α/VEGFA pathway^21^.

In this study we wanted to investigate whether miR-26a-5p has a direct impact on glomerular VEGF expression and leads to impairment of the filtration barrier function.

## Results

### MiR-26a-5p targets VEGF-A in cultured human podocytes trough PIK3C2α

**Figure 1:**
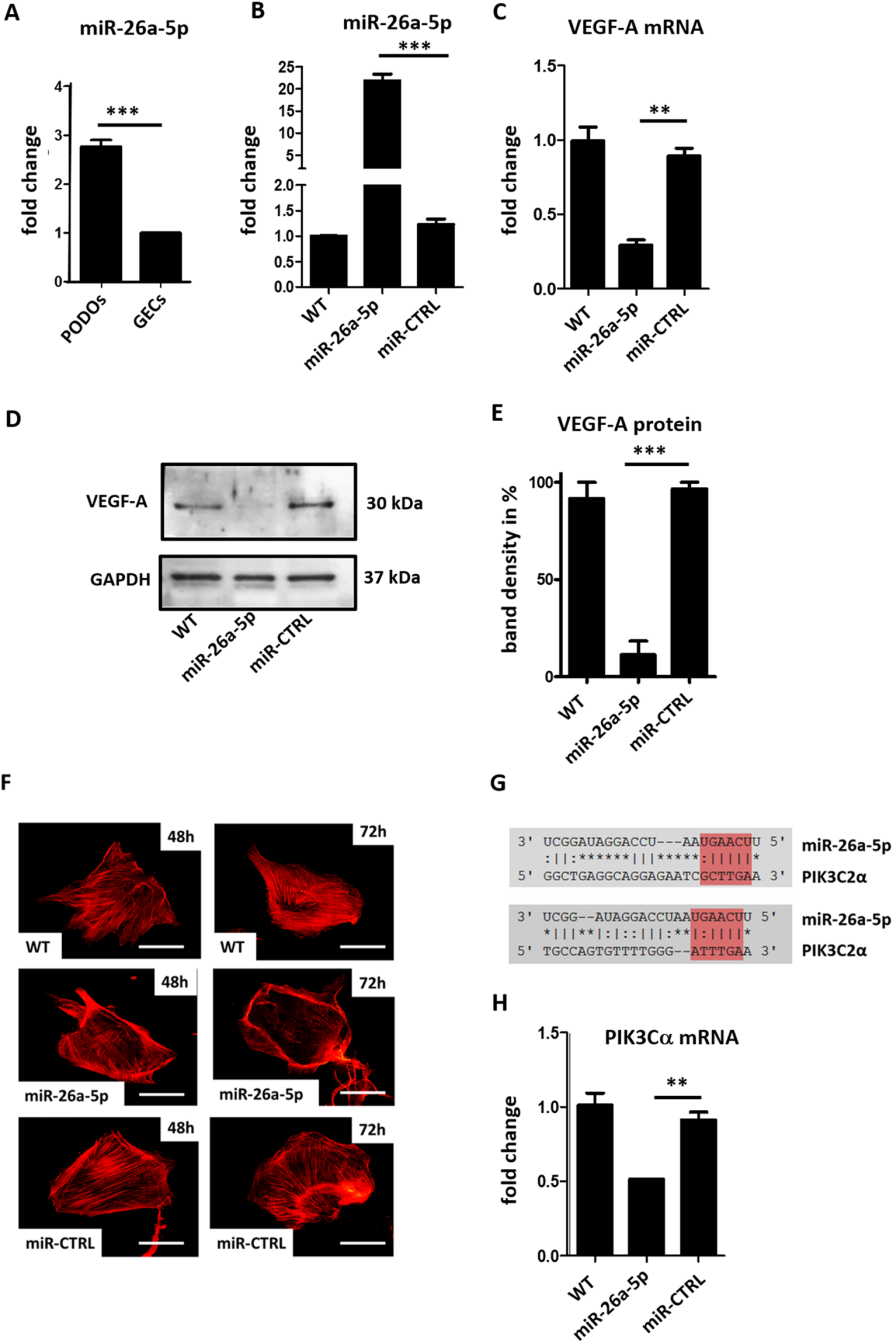
miR-26a-5p regulated VEGF-A in cultured human podocytes. (**A**) Expression of miR-26a-5p in cultured human podocytes (h. PODs) and human glomerular endothelial cells (h. GECs). Expression levels are given as fold change compared to h. GECs. ***p < 0.001. (**B**) TaqMan qPCR for miR-26a-5p in cultured human podocytes 72 h after a 4 h transfection with a miR-26a-5p mimic (miR-26a-5p) compared to a miR control (miR-CTRL) and untransfected condition (WT). ***p < 0.001. (**C**) Fold change in relative VEGF-A mRNA expression in cultured human podocytes 72 h after a 4 h transfection with a miR-26a-5p mimic (miR-26a-5p) compared to a miR control (miR-CTRL) and untransfected condition (WT). **p < 0.001. (**D** Western blot for VEGF-A in cultured human podocytes 72 h after a 4 h transfection with a miR-26a-5p mimic (miR-26a-5p) compared to a miR control (miR-CTRL) and untransfected condition (WT). (**E**) Quantification of western blot bands shown in 1G. Statistic was done with three independent blots shown in supplementary Fig. 2. The band with the highest density of the each blot was set to be 100%. ***p < 0.001. (**F**) Cytoskeleton rearrangement after transfection on cultured human podocytes with a miR-26a-5p mimic (miR-26a-5p) or a control miR mimic (miR-CTRL). Actin fibers were visualised using Alexa Fluor® 546 phalloidin (red). Size bar = 20µm. (**G**) Two different binding sites of miR-26a-5p seed region to PIK3C2α predicted by FINDTAR3. (**H**) Fold change in relative PIK3C2α mRNA expression in cultured human podocytes 72 h after a 4 h transfection with a miR-26a-5p mimic (miR-26a-5p) compared to a miR control (miR-CTRL) and untransfected condition (WT). **p < 0.001.

We examined the expression of miR-26a-5p in cultured human glomerular endothelial cells (h. GECs) and human podocytes (h. PODs). Levels of miR-26a-5p were significantly higher in cultured human podocytes (Fig. 1A).

To validate VEGF-A as a target of miR-26a-5p we transfected cultured human podocytes with a miR-26a-5p mimic. We controlled for successful transfection of the miR mimic by performing TaqMan qPCR for miR-26a-5p in the transfected cells 72 h after transfection (Fig. 1B). Interestingly, overexpression of miR-26a-5p in cultured human podocytes resulted in a 5-fold downregulation of VEGF-A mRNA expression compared to miR control (miR-CTRL) transfected cells (Fig. 1C). Downregulation of VEGF-A protein 72 h after transfection with a miR-26a-5p mimic could be demonstrated with western blot technique (Fig. 1D,E). Transfection of cultured human podocytes with a miR-26a-5p mimic caused a reorganization of the actin cytoskeleton that was time dependent (Fig. 1F).

Using target prediction tools (FindTar3) we first identified the potential binding site of miR-26a-5p in the 3′UTR region of VEGF-A (supplementary Fig. 1A). However, luciferase assay revealed only a marginal effect and did not prove a direct binding of miR-26a-5p to VEGF-A in two different concentration used for miR-26a-5p mimic transfection (supplementary Fig. 1B,C).

Phosphatidylinositol-4-phosphate 3-kinase C2 domain-containing alpha polypeptide (PIK3C2α) is another potential target of miR-26a-5p with even two binding sites. The predicted free energy for these bindings is −20.4 and −17 kcal/mol respectively. According to the assessment criteria for targets predicted by FindTar3 based on central loop score and free energy this binding was considered to be good (Fig. 1G).

With the cloning of wild-type human PIK3C2α 3′UTR to a luciferase reporter construct, we could emphasize that miR-26a-5p directly targets PIK3C2α 3′UTR (supplementary Fig. 1D).

PIK3C2α is an upstream protein in the VEGF-A signaling pathway and the effect of miR-26a-5p on modulating VEGF-A expression through the PIK3C2α/Akt/HIF-1α/VEGFA pathway was previously described and is well documented^21–23^. After transfection of cultured human podocytes with a miR-26a-5p mimic PIK3C2α mRNA was down regulated (Fig. 1H).

Therefore, we conclude that regulation of VEGF-A through miR-26a-5p is most likely indirect via PIK3C2α.

### Injection of a Vegf-Aa specific morpholino or a miR-26a-5p mimic leads to generalized edema in zebrafish

The zebrafish (Danio rerio) homologues of human VEGF-A are vegf-Aa and vegf-Ab^24^. To specifically knockdown each isoforms in zebrafish we injected either a vegf-Aa morpholino (vegf-Aa-MO) or a vegf-Ab-MO in one to four cell stages. The vegf-Aa knockdown by vegf-Aa-MO caused a phenotype with generalized edema 96 hours post fertilization (hpf).

**Figure 2:**
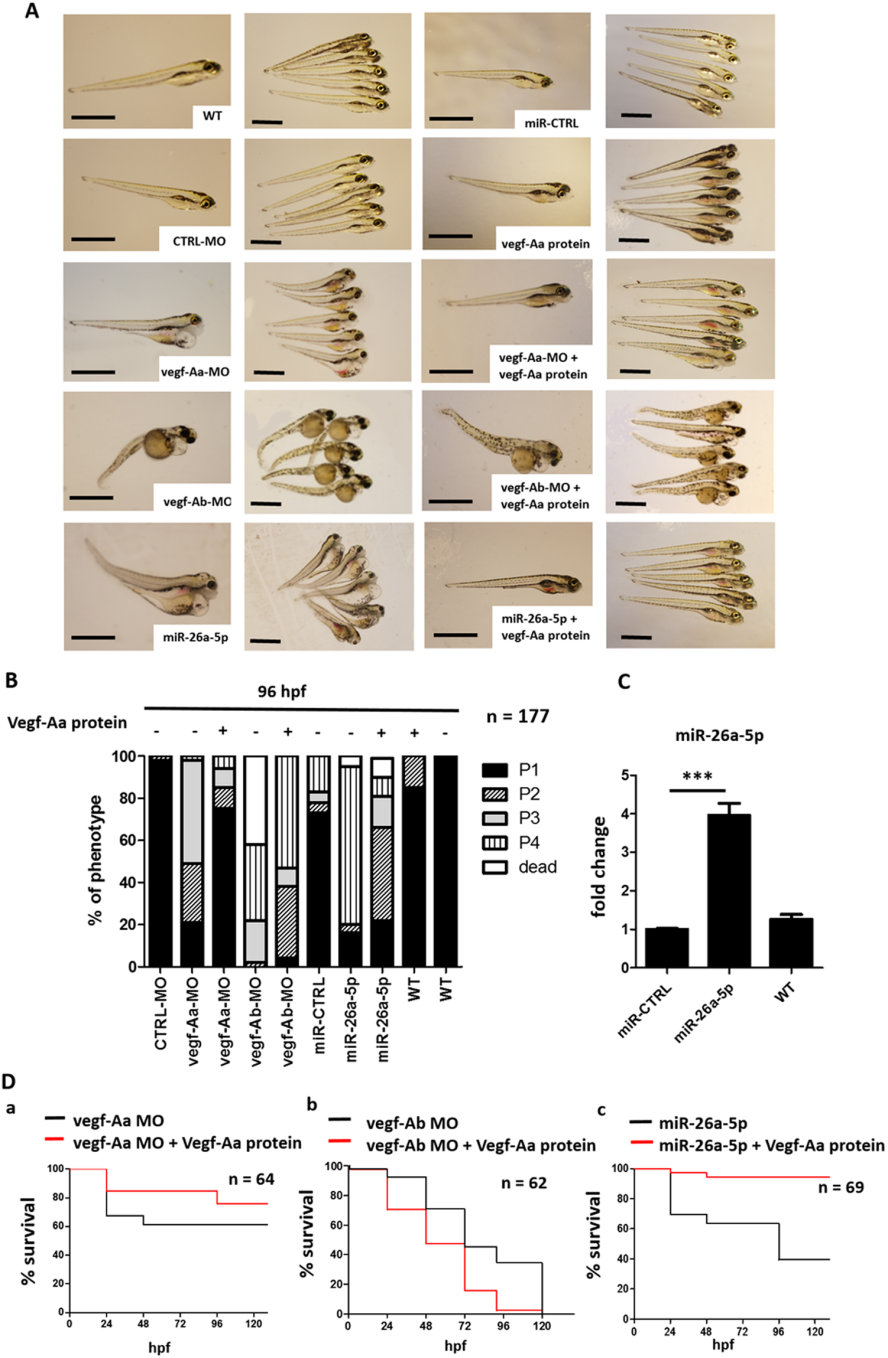
Vegf-Aa knockdown by vegf-Aa-MO or miR-26a-5p mimic leads to edema and loss of plasma proteins. Zebrafish were either injected with control morpholino (CTRL-MO), a vegf-Aa morpholino (vegf-Aa-MO), a vegf-Ab morpholino (vegf-Ab-MO), a control miR mimic (miR-CTRL) or a miR-26a-5p mimic (miR-26a-5p) alone (−) or as co-injection with a recombinant zebrafish Vegf-Aa protein (Vegf-Aa) (+) at one to four cell stages. *p <  0.05, **p <  0.01, ***p < 0.001, n.s. not significant. (**A**) Phenotype pictures of zebrafish larvae at 96 hpf. Scale bar = 500 µM. (**B**) Quantification of edemous phenotype of zebrafish larvae at 96 hpf. The edemous phenotype was categorized in 4 groups: P1 = no edema, P2 = mild edema, P3 = severe edema, P4 = very severe edema, Dead fish are those found dead between 72 hpf and 96 hpf; CTRL-MO n = 15, vegf-Aa-MO n = 20, vegf-Aa-MO + Vegf-Aa n = 21, vegf-Ab-MO n = 16, vegf-Ab-MO + Vegf-Aa n = 16, miR-CTRL n = 16, miR-26a-5p n = 14, miR-26a-5p + Vegf-Aa n = 24, WT + Vegf-Aa n = 17, WT n = 18, total n = 177. (**C**) TaqMan qPCR for miR-26a-5p in whole zebrafish larvae lysate 120 h after injection of a miR-26a-5p mimic (miR-26a-5p) or a miR control (miR-CTRL) in one to four cell stage. ***p < 0.001. n = 12 for each group, total n = 36. (**D**) Survival of zebrafish over time. Survival is given in percent [%] of zebrafish of each group. a) Survival of zebrafish injected with a vegf-Aa morpholino (vegf-Aa-MO) in black versus co-injection of a vegf-Aa morpholino and a Vegf-Aa protein (vegf-Aa-MO + Vegf-Aa protein) in red; n = 64. b) Survival of zebrafish injected with a vegf-Ab morpholino (vegf-Ab-MO) in black versus co-injection of a vegf-Ab morpholino and a Vegf-Aa protein (vegf-Ab-MO + Vegf-Aa protein) in red; n = 62. c) Survival of zebrafish injected with a miR-26a-5p mimic (miR-26a-5p) in black versus co-injection of a miR-26a-5p mimic and a Vegf-Aa protein (miR-26a-5p + Vegf-Aa protein) in red; n = 69.

We quantified edema of zebrafish from P1 to P4 with P1 = zebrafish larvae with no edema and P4 = zebrafish larvae with very severe edema as previously described^25^. 48% of the zebrafish developed P3 edema and 26% P2 edema when injected with a vegf-Aa-MO. Overexpression of miR-26a-5p by injection of a specific miR-26a-5p mimic at the one to four cell stage was still detectable at 120 hpf. (Fig. 2A,B) and caused severe edema comparable to that after vegf-Aa-MO induced knockdown. Injection of the vegf-Ab-MO not only induced edema but also growth retardation and a curved body shape of the fish.

A recombinant zebrafish Vegf-Aa could reduce edema formation when it was co-injected with the vegf-Aa-MO or the miR-26a-5p mimic. However, this protein was not able to rescue the phenotype induced by vegf-Ab knockdown (Fig. 2A,B; *n* = 177). To control for successful overexpression of the miR mimic in the larvae we performed TaqMan qPCR in whole zebrafish lysates at 120 hpf. We could demonstrate that the miR-26a-5p mimic was still detectable at 120 hpf and the expression levels of miR-26a-5p were 4 times higher compared to control at that time (Fig. 2C).

Interestingly, co-injection of the vegf-Aa-MO or the miR-26a-5p mimic with recombinant Vegf-Aa protein significantly increased the survival of zebrafish (62% survival in the vegf-Aa-MO group versus 77% survival in the vegf-Aa-MO + Vegf-A protein group and 40% survival in the miR-26a-5p mimic group versus 94% in the miR-26a-5p mimic + Vegf-A protein group at 120 hpf). Vegf-Aa protein was also able to prolong survival in zebrafish injected with the vegf-Ab-MO until 96 hpf. However, the fish were not able to survive longer than 120 hpf (Fig. 2D).

### Vegf-Aa knockdown by vegf-Aa-MO or miR-26a-5p mimic induces loss of plasma proteins in zebrafish

**Figure 3:**
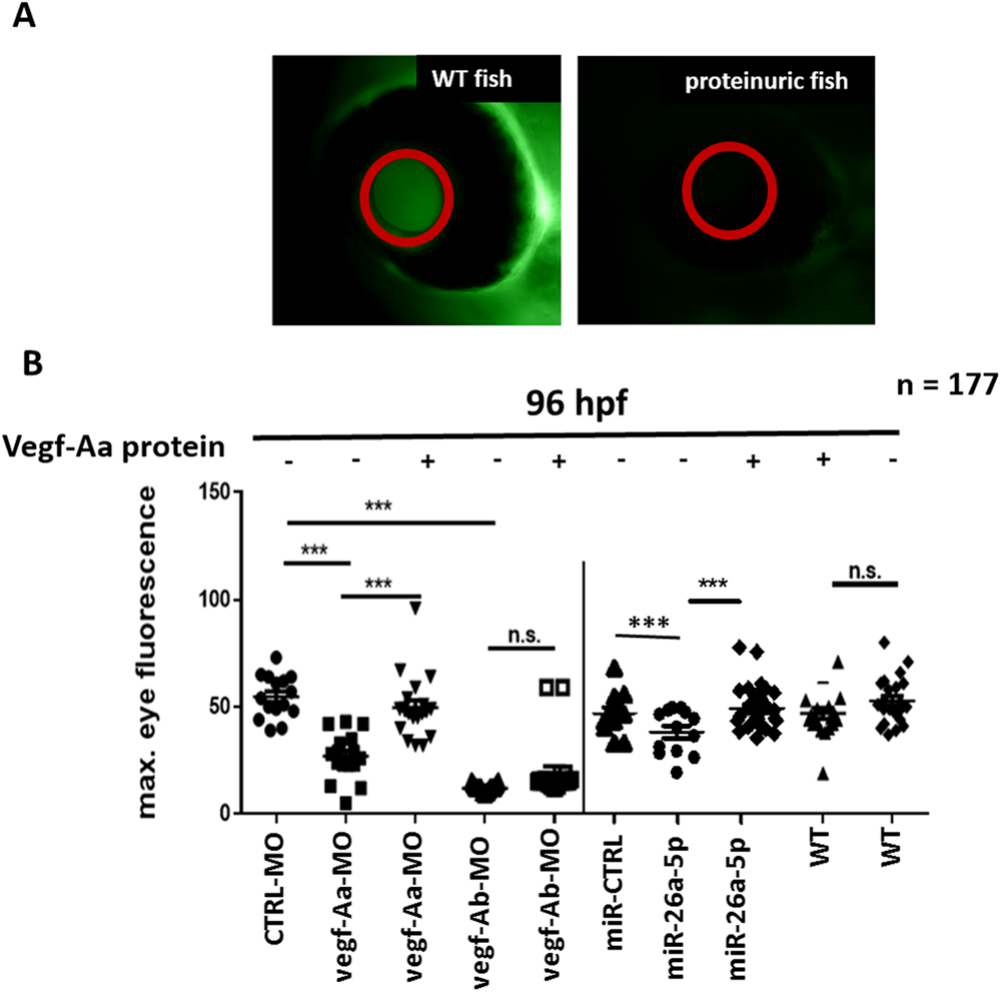
Vegf-Aa knockdown by vegf-Aa-MO or miR-26a-5p mimic causes loss of plasma proteins in zebrafish larvae. Zebrafish were either injected with control morpholino (CTRL-MO), a vegf-Aa morpholino (vegf-Aa-MO), a vegf-Ab morpholino (vegf-Ab-MO), a control miR mimic (miR-CTRL) or a miR-26a-5p mimic (miR-26a-5p) alone (−) or as co-injection with a recombinant zebrafish Vegf-Aa protein (Vegf-Aa) (+) at one to four cell stages. (**A**) Representative images of eye of an unaffected Tg(l-fabp:DBP:eGFP) transgenic fish appears green due to the fluorescent plasma protein in the retina vessels (left). In fish with proteinuria, the eye appears dark due to loss of green fluorescent plasma proteins (right). (**B**) Quantification of loss of circulating high molecular weight proteins by measuring max. eye fluorescence in the retinal vessel plexus of Tg(l-fabp:DBP:eGFP) zebrafish larvae at 96 hpf.***p < 0.001, n.s. not significant; CTRL-MO n = 15, vegf-Aa-MO n = 20, vegf-Aa-MO + Vegf-Aa n = 21, vegf-Ab-MO n = 16, vegf-Ab-MO + Vegf-Aa n = 16, miR-CTRL n = 16, miR-26a-5p n = 14, miR-26a-5p + Vegf-Aa n = 24, WT + Vegf-Aa n = 17, WT n = 18, total n = 177.

To test for protein leakage through the glomerular filter, we used transgenic zebrafish that express a Vitamin D binding protein tagged with enhanced green fluorescent protein (eGFP) under the control of the liver-type fatty acid binding protein (l-fabp) promoter as previously described^25–27^. The DBP-eGFP fusion protein has a molecular weight of approximately 78 kDa and can easily be detected in the retinal vessel of the zebrafish. Loss of eGFP, quantified over the retinal vessel plexus, indicates loss plasma proteins through glomerular leakage (Fig. 3A).

Vegf-A knockdown by injection of either a vegf-Aa-MO, a vegf-Ab-MO or a miR-26a-5p mimic at one to four cell stages caused a significant loss of plasma proteins at 96 hpf. Co-injection of the recombinant zebrafish Vegf-Aa was able to rescue the loss of plasma proteins caused by vegf-Aa-MO and miR-26a-5p mimic but was not able to significantly decrease the proteinuria caused by vegf-Ab-MO (Fig. 3B; *n* = 177). This is also partially explained due to the poor survival in the vegf-Ab-MO group.

### Impaired intersegmental tail vessel sprouting after vegf-Aa knockdown by vegf-Aa-MO or miR-26a-5p mimic in zebrafish

**Figure 4:**
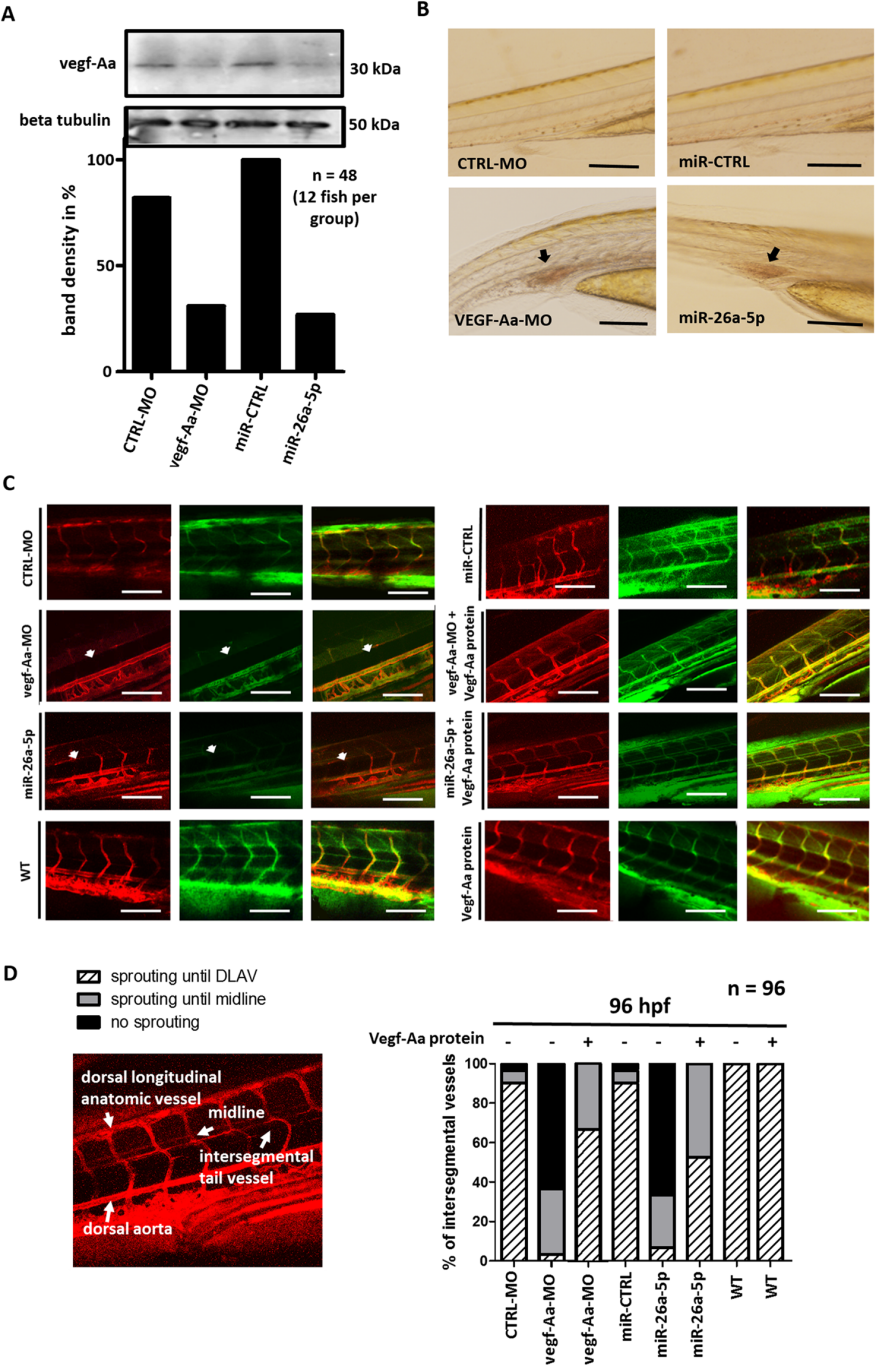
Vegf-Aa knockdown by vegf-Aa-MO or miR-26a-5p mimic cause impairments in intersegmental tail vessel sprouting. (**A**) Western blot for Vegf-Aa and normalization with Gapdh of whole zebrafish larvae lysate (10 µg of a pool of 10 fish). Zebrafish were injected with either a recombinant zebrafish Vegf-Aa protein (Vegf-Aa protein), a control morpholino (CTRL-MO), a vegf-Aa morpholino (vegf-Aa-MO), a control miR mimic (miR-CTRL) or a miR-26a-5p mimic (miR-26a) at the one to four cell stages. Zebrafish were harvested at 96 hpf. Quantification of western blot bands are shown below the blot. n = 12 fish per group, total n = 48. Full-length blots are presented in supplementary Fig. 3. (**B**) Phenotype pictures of zebrafish show blood pooling in tail region (arrow heads) in zebrafish that were injected with a vegf-Aa morpholino (vegf-Aa-MO) or a miR-26a-5p mimic (miR-26a-5p) were as control injected fish (CTRL-MO and miR-CTRL) appeared normal. Scale bar: 4 mM. (**C**) Pictures of tail vessel region of Tg(l-fabp:DBP-eGFP/flk-mcherry) zebrafish. These transgenic fish express a red fluorescent endothelium and green fluorescent plasma protein. Zebrafish were injected with either a control morpholino (CTRL-MO), a vegf-Aa morpholino (vegf-Aa-MO) a miR-26a-5p mimic (miR-26a-5p), a control miR mimic (miR-CTRL) alone (−) or together with a recombinant zebrafish Vegf-Aa protein (Vegf-Aa) (+) at the one to four cell stages. Vessel pictures were taken at 96 hpf. Arrow heads illustrate defects in sprouting of intersegmental vessel in vegf-Aa-MO and miR-26a-5p mimic injected fish. DLAV: dorsal lateral anatomic vessel. Scale bar = 500 µM. (**D**) Representative tail vessel pictures of Tg(l-fabp:DBP-eGFP/flk-mcherry) zebrafish with normal intersegmental tail vessel sprouting (left) and quantification of intersegmental vessels sprouting in zebrafish that were injected with miR mimics and morpholinos as indicated (right). n = 12 per group, total n = 96.

To confirm that Vegf-Aa protein level was reduced in the zebrafish after both vegf-Aa-MO and miR-26a-5p mimic injection, we performed western blot analysis of whole zebrafish larvae (10 per group) at 96 hpf. The analysis with a zebrafish specific vegf-Aa antibody confirmed that Vegf-Aa protein was downregulated after vegf-Aa-MO and miR-26a-5p mimic injection (Fig. 4A).

Interestingly, about 50% of our zebrafish displayed reduced or absent blood flow in their distal tail region after vegf-Aa knockdown. When we closely examined the tail vessels we could detect significant blood pooling in zebrafish injected with the vegf-Aa-MO or miR-26a-5p mimic. This blood pooling was never detected in wild type or control injected fish (Fig. 4B).

To study the zebrafish tail vasculature further we used the Tg(l-fabp:DBP-eGFP/flk-mcherry) fish with red fluorescent endothelium and green fluorescent plasma protein as previously described^25^. Intersegmental tail vessels normally sprout from the dorsal aorta towards the dorsal neural tube (DNT) where, upon approaching the DNT, they connect with other intersegmental vessels to form the dorsal longitudinal anastomotic vessel. We investigated vessel formation after injection of MO or miR-mimics at 96 hpf. In wild type and control injected zebrafish, tail vessel sprouting was normal. Impairments in tail vessel sprouting after vegf-Aa knockdown by MO resembled those after miR-26a-5p mimic injection (Fig. 4C). 63% of vegf-Aa-MO and 65% of miR-26a-5p mimic injected fish did not develop intersegmental tail vessels. Only 3% of vegf-Aa-MO and 7% of miR-26a-5p mimic injected fish showed complete intersegmental tail vessel sprouting. These impairments could be ameliorated by a co-injection of a zebrafish Vegf-Aa protein (64% normal intersegmental vessel sprouting in vegf-Aa-MO/Vegf-Aa protein co-injected zebrafish and 53% normal intersegmental vessel sprouting in miR-26a-5p-mimic/Vegf-Aa protein co-injected zebrafish) (Fig. 4C,D).

### Vegf-Aa knockdown by miR-26a-5p causes proteinuria and edema in 48 hpf developed zebrafish

**Figure 5:**
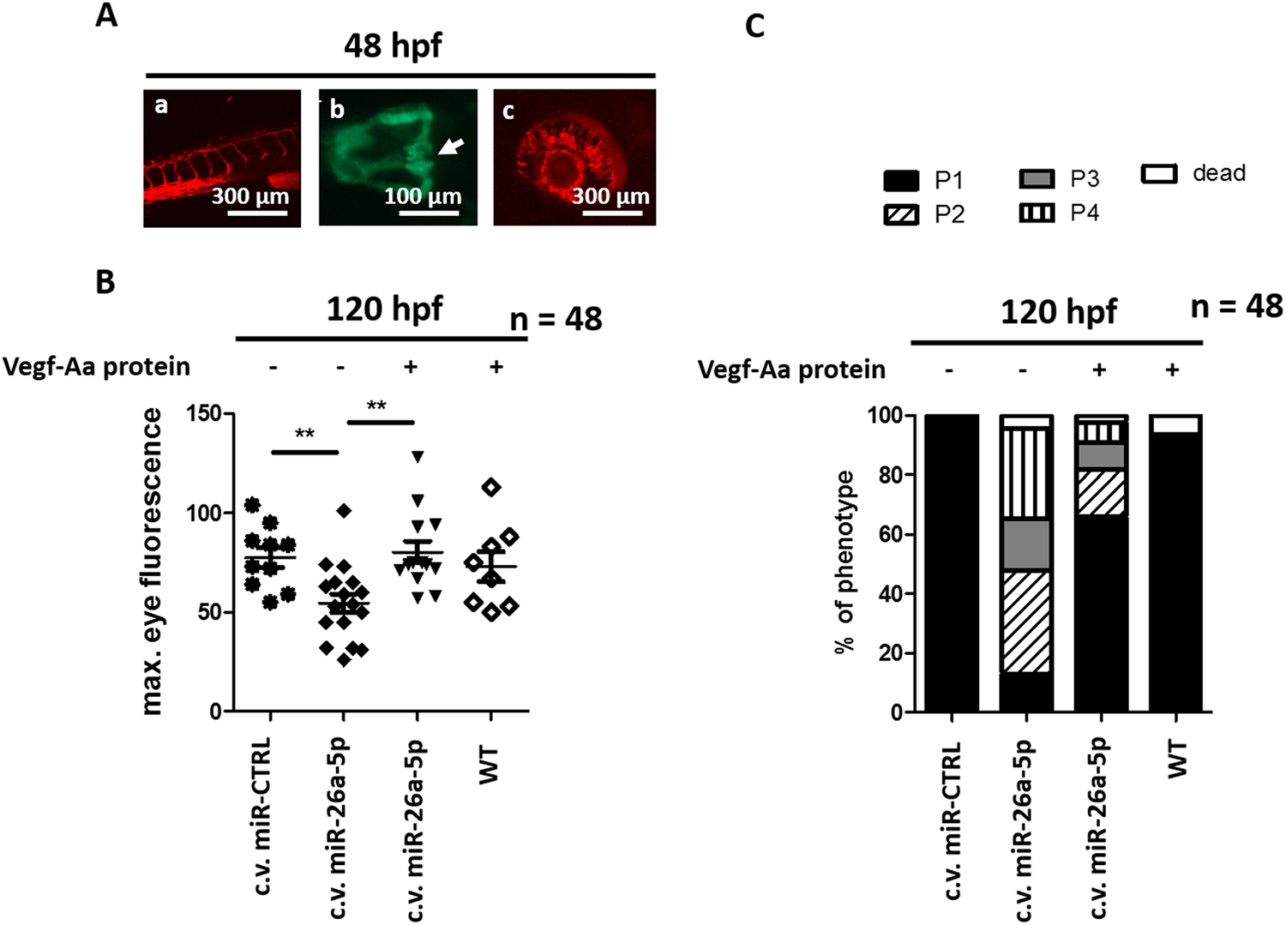
Vegf-Aa knockdown by miR-26a-5p causes proteinuria and edema in afore healthy zebrafish (**A**): Pictures of tail vessels (a), glomeruli fusing at zebrafish midline (b) and eye vessel plexus (c) of 48 h old zebrafish shows proper vascular development as well as glomerular fusion. Tail and eye vessel pictures were taken of a Tg(l-fabp:DBP-eGFP/Flk-mcherry) transgenic zebrafish with red fluorescent endothelium. Glomerular fusion is shown in a Tg(wt1b:eGFP) transgenic zebrafish that expresses eGFP under control of the wt1b promotor, arrow points at the fused glomerulus). Scale bar = 100 µM and 300 µM as indicated. (**B**,**C**) Quantification of loss of plasma proteins by measuring maximum eye fluorescence of the retinal vessel plexus (**B**) and quantification of edemous phenotype (**C**) of Tg(l-fabp:DBP:eGFP) zebrafish larvae at 120 hpf. A control miR mimic (miR-CTRL), a miR-26a-5p mimic (miR-26a-5p) or the combination of miR-26a-5p and Vegf-Aa protein were injected in the cardinal vein at 48 hpf. At this time the tail vasculature, glomerular fusion and retinal vessel formation was already formed; c.v. miR-CTRL n = 10, c.v. miR-26a-5p n = 17, miR-26a-5p + Vegf-Aa n = 13, WT + Vegf-Aa n = 8, total n = 48.

The above described effects on the zebrafish vasculature could also indicate developmental defects in vegf-Aa-MO and miR-26a-5p mimic injected zebrafish. To rule out that impairments in glomerular or vascular maturation affected our assay for proteinuria measurement in zebrafish, we also performed miR-26a-5p overexpression experiments in zebrafish larvae with an already developed vascular and pronephros system. To accomplish this we performed an injection of a miR-26a-5p mimic in the cardinal vein (c.v.) of anesthetized zebrafish at 48 hours post fertilization (hpf). The literature describes the proper development of the pronephros in wild type animals with fusion of two glomeruli at 48 hpf^28,29^. Prior to our c.v. injection we checked for normal vascular sprouting in the eye and tail region of the Tg(l-fabp:DBP-eGFP/flk-mcherry) fish and for proper glomerular fusion in Tg(wt1b:eGFP) fish that express eGFP under control of the wt1b promotor (Fig. 5Aa–c). Similar to earlier experiments, injection of a miR-26a-5p mimic in the zebrafish larvae at this later time point led to loss of plasma proteins and edemous phenotype. 47% of zebrafish with c.v. vegf-Aa-MO injection developed P3 or P4 edema. Again, these findings could be rescued if the miR-26a-5p mimic was co-injected with a recombinant zebrafish Vegf-Aa protein in the c.v. at 48 hpf. Only 16% of zebrafish showed P3 or P4 edema after a co-injection of the miR-26a-5p mimic together with the Vegf-Aa protein, indicating that vegf-Aa downregulation was the causative factor for inducing proteinuria in 48 hpf developed zebrafish larvae as well (Fig. 5B,C; n = 80).

### Vegf-Aa knockdown by vegf-Aa-MO or miR-26a-5p mimic leads to glomerular endotheliosis and podocyte effacement that can be rescued by recombinant zebrafish Vegf-Aa protein

**Figure 6:**
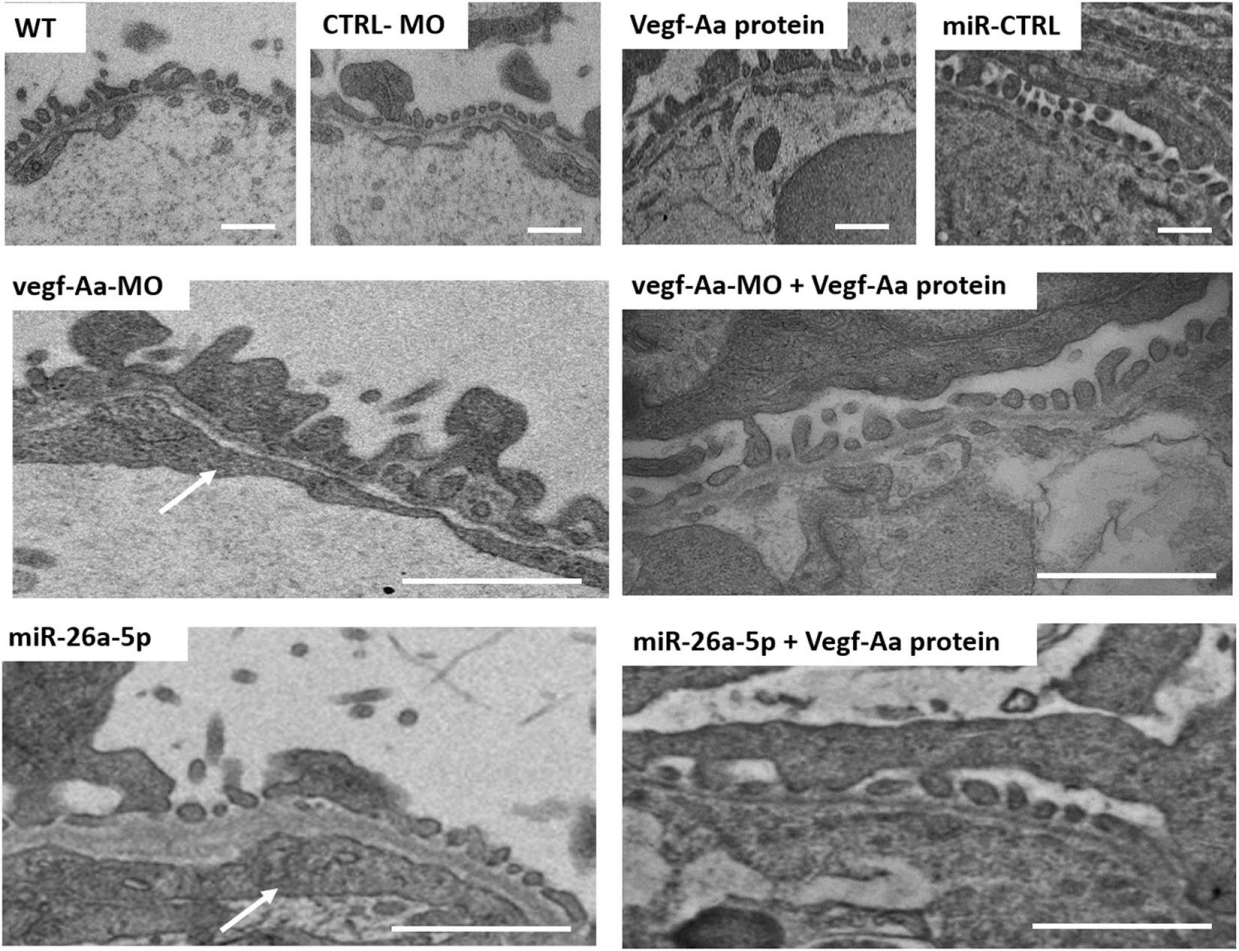
Vegf-Aa knockdown by vegf-Aa-MO or miR-26a-5p mimic causes glomerular endotheliosis and podocyte effacement that can be rescued by Vegf-Aa protein. Transmission electron microscopy pictures showing glomerular endotheliosis and podocyte effacement vegf-Aa knockdown by injection of a vegf-Aa morpholino (vegf-Aa-MO) or a miR-26a-5p mimic (miR-26a-5p). These pathologies were ameliorated when a zebrafish Vegf-Aa protein was co-injected. Pictures of the glomerulus were taken of 120 h old fish. Zebrafish were injected with a control morpholino (CTRL-MO), a vegf-Aa morpholino (vegf-Aa-MO), a recombinant zebrafish Vegf-Aa protein (Vegf-Aa protein), a miR-26a-5p mimic (miR-26a-5p), a control miR mimic (miR-CTRL) or a combination of vegf-Aa-MO and recombinant Vegf-Aa protein (vegf-Aa-MO + Vegf-Aa) a.e a combination of miR-26a mimic and zebrafish Vegf-Aa protein (miR-26a-5p + Vegf-Aa) as indicated at one to four cell stages or at 48 hpf (c.v. miR-26a, c.v. miR-26a-5p + Vegf-Aa). Scale bar = 500 nm.

To examine which part of the glomerular filtration barrier was affected by our interventions, we performed ultrastructural analysis of the zebrafish glomeruli using transmission electron microscopy. Vegf-Aa knockdown by either injection of a vegf-Aa-MO or a miR-26a-5p mimic led to a swollen endothelium with loss of glomerular endothelial cell-specific fenestration and podocyte effacement. The glomeruli of control injected zebrafish as well as Vegf-Aa protein injected zebrafish showed a preserved normal podocyte and glomerular endothelium structure comparable to uninjected wildtype fish. Zebrafish Vegf-Aa protein was able to ameliorate glomerular damage induced by vegf-Aa-MO and miR-26a-5p mimic (Fig. 6). Vegf-Ab-MO injected zebrafish were not investigated on ultrastructural level since we had no significant survival at 120 hpf.

### MiR-26a-5p is expressed in urine samples from preeclamptic patients with podocyturia

**Figure 7:**
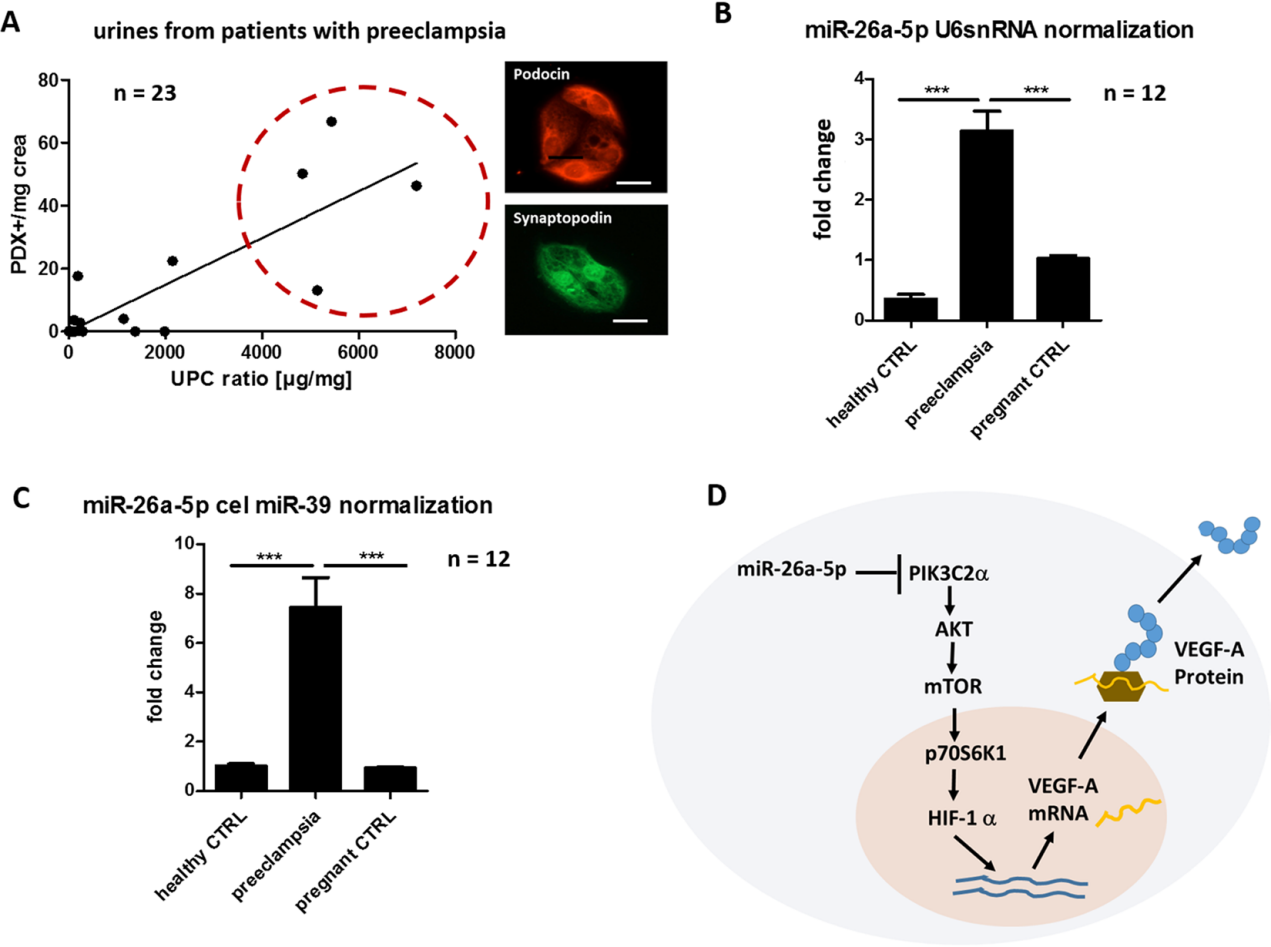
MiR-26a-5p is upregulated in urines from patients with preeclampsia and podocyturia. (**A**) Correlations between proteinuria measured in urine protein creatinine ratio (UPC-ratio; [µg/mg]) and podocyturia measure in podocalyxin positive cells in the overnight cultured urine sediment per urinary creatinine [PDX+ cells/mg crea] in patients with preeclampsia. n = 23. (**B**) Fold change in miR-26a-5p expression in urine samples from healthy non pregnant controls and patients with preeclampsia compared to urine samples from healthy non pregnant controls (baseline of 1). MiR expression was normalized with endogenous U6snRNA. Total n = 12. (**C**) Fold change in miR-26a-5p expression in urine samples from healthy non pregnant controls and patients with preeclampsia compared to urine samples from healthy non pregnant controls (baseline of 1). MiR expression was normalized with cel-miR-39. (**D**) Schematic illustration of how miR-26a-5p downregulates VEGF-A through PIK3C2α. PIK3C2α**:** Phosphatidylinositol-4-phosphate 3-kinase C2 domain-containing alpha polypeptide.

In a small pilot study with 23 patients with preeclampsia we looked for proteinuria and podocyturia. We detected podocyturia by staining vital cells from overnight cultured urine sediments for podocalyxin (PDX) as previously described^30–33^. With this method we could detect vital podocytes in urines from 11 patients (48%). We quantified proteinuria with urine protein creatinine ratio (UPC-ratio in µg/mg) and podocyturia with PDX+ cells in the urine sediment per urine creatinine (PDX+/mg crea). Podocyturia correlated with proteinuria (r = 0.84). Mean podocyturia was 10.32 PDX+ cells/mg crea in all patients and 20.65 PDX+ cells/mg crea if only patients with podocyturia were included (Fig. 7A, Table 1).

**Table 1:** Characteristics of preeclamptic patients and controls.

Diagnosis	Age [years]	UPC-ratio [µg/mg]	Podocyturia [PDX+/mg crea]
**PREEC**	**38**	**5431.72**	**66.76**
**PREEC**	**35**	**4834.43**	**50.26**
**PREEC**	**41**	**7195.29**	**46.38**
**PREEC**	**30**	**2144.00**	**22.44**
PREEC	34	184.80	17.60
PREEC	38	5141.71	13.07
PREEC	37	1131.43	4.02
PREEC	30	101.54	3.61
PREEC	28	231.26	2.78
PREEC	29	12.94	0.21
**Mean of PREEC with podocyturia**	**34**	**2525.55**	**20.65**
PREEC	31	1980.00	0.00
PREEC	31	1371.93	0.00
PREEC	35	283.61	0.00
PREEC	28	135.77	0.00
PREEC	31	112.82	0.00
PREEC	33	80.58	0.00
PREEC	23	42.48	0.00
PREEC	38	16.34	0.00
PREEC	34	8.80	0.00
PREEC	29	4.11	0.00
PREEC	30	14.78	0.00
PREEC	25	19.94	0.00
PREEC	28	24.98	0.00
**Mean of PREEC without podocyturia**	**32**	**1326.32**	**10.32**
pregnant CTRL	32	negative	not detected
pregnant CTRL	28	negative	not detected
pregnant CTRL	34	negative	not detected
pregnant CTRL	37	negative	not detected
pregnant CTRL	32	negative	not detected
**Mean of pregnant CTRL**	**33**	**negative**	**not detected**
Non-pregnant CTRL	38	negative	not detected
Non-pregnant CTRL	32	negative	not detected
Non-pregnant CTRL	33	negative	not detected
Non-pregnant CTRL	38	negative	not detected
Non-pregnant CTRL	30	negative	not detected
**Mean of non-pregnant CTRL**	**34**	**negative**	**not detected**

Age, urine protein creatinine ratio (UPC-ratio; [µg/mg]) and podocyturia (podocalyxin positive cells in the overnight cultured urine sediment per urinary creatinine [PDX+ cells/mg crea]) in patients with preeclampsia (PREEC, n = 23), pregnant control (pregnant CTRL, n = 5) and non-pregnant control (non-pregnant CTRL, n = 5). The PREEC patients with highest podocyturia were used for miR experiments in Fig. 7B,C.

Next, we looked for miR-26a-5p expression in urine samples from patients with preeclampsia compared to healthy non-pregnant women and healthy pregnant controls. We only included urines from preeclamptic patients with high podocyturia (>20.65 PDX cells/mg crea) and high proteinuria (UPC-ratio >2 µg/mg) to only consider those patients with ongoing podocyte damage (first four patients in Table 1).

We spiked-in control cel-miR-39 to consider the experimental influences on the samples. By generating fold changes in urinary miR-26a-5p expression in patients with preeclampsia compared to non-pregnant and pregnant healthy controls we could show that miR-26a-5p expression was significantly elevated in urines from preeclamptic women with ongoing podocyte damage (Fig. 7B). We also used U6 snRNA as an endogenous normalization for our urinary miR data and confirmed upregulation of miR-26a-5p in preeclampsia (Fig. 7C).

As RNU6B, a small noncoding RNA which is exclusively expressed in nuclei, was not detectable in our urine samples we could prove that our urine supernatant was cell free (data no shown).

Figure 7D summarizes the pathway how miR-26a-5p downregulates VEGF-A though PIK3C2α.

## Discussion

Preeclampsia is a leading cause of morbidity and mortality in pregnancy. Alterations of the angiogenic pathways in early gestation are thought to contribute to the inadequate cytotrophoblast invasion in the placenta characteristic of preeclampsia^34^. One of the main hypothesis of the pathophysiology of preeclampsia is that an elevated sFLT1 level binds and inhibits VEGF-A^4,35^. However, there is some controversy regarding the role of the VEGF system in preeclampsia. A decrease in placental VEGF-A has been reported in different studies^36–38^. However, it has been argued that the VEGF-A: sFLT1 ratio seen in preeclampsia is not the entire story; sFLT1 levels are not high enough to explain the absolute reduction in circulating VEGF-A seen in preeclampsia^39,40^.

Furthermore, glomerular changes seen in preeclampsia remain to some extent puzzling. Glomerular VEGF-A is produced by podocytes and acts in a paracrine manner on endothelial cells. Thus, the glomerulus is more dependent on local VEGF-A than on circulating VEGF-A levels. The molecular weight of circulating sFLT1 is around 85–120 kDa^41^. To reach the podocytes sFLT1 would need to cross the glomerular filtration barrier, which in term of its scale should not be possible without a preexisting glomerular damage. Thus, it is not completely understood how sFLT1 can inhibit glomerular VEGF-A.

MiRs that act by inhibiting target mRNA translation, have increasingly been implicated in the pathology of a number of diseases^14^. Preeclampsia related miRs have been found in serum and their targets have been studied in placenta tissue and serum^16,17,42,43^.

MiR-26a-5p was upregulated in placentas and plasma from preeclamptic women^20^. In another context, miR-26a-5p has also been described to regulate VEGF-A expression in hepatocellular carcinoma^21^. Therefore, we hypothesize that miR-26a-5p is involved in glomerular pathology seen in preeclampsia and base our observation on the following lines of evidence:

In the glomerulus, miR-26a-5p is predominantly expressed by podocytes^44,45^. We demonstrated that exogenous miR-26a-5p decreases VEGF-A expression in cultured human podocytes. Even though there is a predicted direct binding side of miR-26a-5p to VEGF-A a reporter luciferase assay for the functional assessment of this interaction could not prove a direct binding. These data indicate that the effect of miR-26a-5p on modulating VEGF-A expression is again indirectly regulated via the PIK3C2α/Akt/HIF-1α/VEGFA pathway as previously described in HCC^21–23^. Thus, miR-26a-5p has a regulatory function on the local VEGF-A in the glomerulus, where podocytes are the major source of VEGF-A. Moreover, MiR-26a-5p expression also effected the actin cytoskeleton of cultured podocytes directly (Fig. 1F). It was previously described, that VEGF-A induces endothelial cell migration and is involved in actin polymerization, and focal adhesion assembly^46–48^.

VEGF-A also stimulated remodeling of the actin cytoskeleton into stress fibers in microvascular endothelial cells and HUVECs^49,50^.

We used the zebrafish model to study functional and ultrastructural abnormalities of the glomerular filtration barrier due to miR-26a-5p overexpression. Since zebrafish share a high degree of genomic sequence and protein homology with humans and the zebrafish pronephros consists of a single glomerulus that is structurally very similar to its human counterpart, it serves as an ideal model for glomerular diseases^51^. Fluorescent dextran injection experiments demonstrate that the onset of zebrafish glomerular filtration occurs around 48 hpf^52^.

Overexpression of miR-26a-5p in zebrafish by microinjection of a specific miR-26a-5p mimic was sufficient to resemble glomerular changes of preeclampsia^1^ with proteinuria, edema, glomerular endotheliosis with endothelial cell swelling, loss of glomerular endothelial fenestration and podocyte foot process effacement. A recombinant zebrafish Vegf-Aa protein was able to rescue the phenotype caused by vegf-Aa-MO or miR-26a-5p mimic but was not able to significantly rescue vegf-Ab knockdown. Thus, a tight regulation of vegf-A is necessary for maintenance of the zebrafish glomerular filter function. Vegf-A is also known to play a crucial role in angiogenesis^53,54^. Indeed, we detected impairments in intersegmental tail vessel sprouting when we injected a vegf-Aa-MO or miR-26a-5p mimic in zebrafish at the one to four cell stages.

To rule out impairments in angiogenesis or glomerular development as potential confounder in our zebrafish model, we overexpressed miR-26a-5p at a time when angiogenesis and glomerular function is considered largely established. An injection of a miR-26a-5p mimic in the c.v. of the zebrafish at 48 hpf caused proteinuria and edema which could be rescued if the miR-26a-5p mimic was co-injected with a recombinant zebrafish Vegf-Aa protein.

From our data in zebrafish it is tempting to posit that miR-26a-5p is involved in glomerular pathology seen in preeclampsia.

Renal biopsies are rarely performed in pregnant woman. However, a reliable marker for ongoing podocyte damage in preeclampsia is podocyturia^32,55,56^. We looked for miR-26a-5p expression in urine samples from preeclamptic patients with proteinuria and podocyturia and compared the expression levels with those in urine samples from healthy on-pregnant controls as well as healthy pregnant controls in a small pilot study.

Three different normalization strategies for miR qPCR experiments have been described but until now, there is no consensus regarding the normalization of miRs isolated from body fluids. One way of normalization is by using the global mean miR expression^57^. As this method requires a large amount of miRs per sample we did not use the method. A second method normalizes the amount of miR against a spike-in control and considers the experimental influences on the samples^58^. We spiked our urine samples with cel-miR-39 before miR isolation. A third alternative for normalization is the endogenous control method that determines the relative expression of the target gene using abundant and stably expressed endogenous miRs^59^.

In previous miR screening experiments U6 snRNA was very stable expressed not only in urines from control and patients with different glomerular diseases but also in different cultured human glomerular cell lines^45^. Therefore, we used U6 snRNA as an endogenous normalization for our urinary and cell miR data.

Endogenous normalization methods revealed that miR-26a-5p was upregulated in urine samples from patients with preeclampsia compared to healthy non-pregnant and healthy pregnant controls.

To the best of our knowledge, preeclamptic urines have never been screened for miR expression before. MiR-26a-5p was described to be upregulated in plasma and placental tissues of women with severe preeclampsia in another study^60^. Therefore, miR-26a-5p detected in the urine from patients with preeclampsia might have been derived from the placenta, been delivered to the circulation and been filtered into the urine in the glomerulus. However, we also could demonstrate that podocytes express miR-26a-5p and we could detect a downregulation of VEGF-A due to miR-26a-5p in cultured human podocytes. Furthermore, urinary miR-26a-5p was detectable together with podocyturia in urines from preeclamptic women with ongoing podocyte damage. Therefore, it is conceivable that local overexpression of miR-26a-5p by podocytes together with circulating miR-26a-5p derived from the placenta contribute to the reduced glomerular VEGF-A level in preeclampsia. Still, urinary miR-26a-5p expression in preeclampsia has to be confirmed larger patient cohorts.

In summary, we demonstrate that the zebrafish can serve as a model to investigate glomerular injury in preeclampsia. MiR-26a-5p is upregulated in preeclampsia and targets podocyte VEGF-A. The functional and ultrastructural correlates of glomerular changes seen after miR-26a-5p overexpression in zebrafish with proteinuria, edema, glomerular endotheliosis and podocyte effacement highly resembled the finding in human preeclampsia. As miR-26a-5p was increased in our pilot study in urines from patients with preeclampsia and podocyturia, this miR might have the potential to serve as a promising non-invasive biomarker or potential therapeutic target to antagonize the reduction of VEGF-levels in the disease. These should be confirmed in a larger prospective cohort.

## Methods

### MiR detection in cell free urine samples from patients with preeclampsia and in cultured cells

Morning urine was collected from healthy volunteers, healthy pregnant women and from patients with clinical preeclampsia. Active glomerular disease in these patients was defined as having proteinuria more than 3.5 mg/g creatinine. Urine samples (50 ml) were centrifuged at 15.000 rpm for 15 min to pellet the cells and cellular debris. Purification of total RNAs including miRs from cells and cell-free urine was done with miRNeasy Kit (QIAGEN, Venlo, Netherlands) according to the manufacture’s protocol. Purification of total RNAs including miRs from cells and cell-free urine was done with miRNeasy Mini Kit (QIAGEN, Venlo, Netherlands) according to the manufacture’s protocol. For urine samples 5 µl of 5 nM synthetic cel-miR-39 was added to control for variations during preparation and later normalization for endogenous miRs.

For miR reverse transcription and real-time PCR we used TaqMan® MicroRNA Assays (Life Technologies, Carsbad, CA) with miR-specific primers (according to the manufacturer’s protocols. Program for reverse transcription with 5 ng RNA, miR specific revers transcription primer and master mix was run according to the manufactory protocol was 30 min 16 °C, 30 min 42 °C and 5 min 85 °C. TagMan® real-time PCR parameters were the following: 10 min at 95 °C and 40 cycles of 15 seconds at 95 °C following 1 minute at 60 °C.

MiR detection after miR mimic transfection in cultured human podocytes was done according to the miR detection in urines without the spike in step of cel-miR-39.

### Luciferase assay

Luciferase reporter assay to validate miR-26a-5p binding to human PIK3C2A and VEGF-A was done in HEK cells applying the luciferase assay aystem (Promega, E1500, Madison, WI, USA.). At first, primers to amplify 3′-UTR of VEGF-A and PIK3C2A were designed, carrying 5′-SpeI and 3′-HindIII restriction sites to ligate amplicon of target 3′-UTR into the pmirReport vector. Primer design for VEGF-A 3′UTR were forward 5′-AAA ACT AGT ACAGAGAGACAGGGCAGGAT-3′ and reverse 5′-AAA AAG CTT TGCACTAGAGACAAAGACGTGA-3′. Primers to amplify 3′-UTR of PIK3C2A were designed, carrying 5′-SpeI and 3′-HindIII restriction sites to ligate amplicon of target 3′-UTR into the pmirReport vector. Primer design was PIK3C2A_3′UTR_fwd: AAA ACT AGT AGAATCGCTTGAACCCAGGA and PIK3C2A_3′UTR_rev: AAA AAG CTT ACGACCTCTACCAAGACAGT. MiR-26a-5p mimic (30 nM, 50 nM and 100 nM), pmiR-report-3′-UTR and beta-Gal normalizing plasmid (20 ng) were transfected to HEK293 cells. Cells were lysed 24 h after transfection and subsequently used for luciferase activity and beta-Gal activity measurement applying Synergy HT reader (Biotek, Germany). Luciferase reads are normalized with beta-Gal reporter expression values according to manufacturers’ instructions (beta-Gal kit system, Promega, E1500, Madison, WI, USA).

### Detection of podocytes in urine samples from patients with preeclampsia

Spot urine samples of patients were centrifuged at 1200 rpm for 8 min, the supernatant was removed and the pellet was resuspended in a sterile HDF solution (137 mM NaCl, 5 mM KCl, 5.5 mM glucose, 4 mM NaHCO and 0.2% EDTA). After additional centrifugation at 1200 rpm for 8 min, the pellet was resuspended in DMEM/F-12 medium containing 10% FCS, 0.5 U/l penicillin and 0.5 mg/dl streptomycin. The resuspended pellet was seeded equally in a 24-well dish containing collagen I coated cover slides. Samples were incubated at 37 °C with 5% CO overnight. Slides were then fixed at −20 °C for 10 min using ice-cold methanol and permeabilized using 0.1% Triton. After blocking with 10% donkey, serum immunofluorescent staining was done with primary antibodies overnight at 4 °C followed by incubation in secondary antibody for 1 h at room temperature. Finally, slides were mounted on glass slides using Vecta Shield with DAPI (Vector laboratories, Burlingame, CA, USA). Primary antibody was goat anti-podocalyxin (AF1658, R&D systems, MAB1247, Minneapolis) and secondary antibody was donkey anti-goat IgG Cy 3 (Jackson Immuno Research, West Grove, PA, USA).

### Transfection of miR-26a-5p in cultured human podocytes

Culture conditions of conditionally immortalized human podocytes: Podocytes were proliferated under permissive conditions at 33 °C. When cultivated at 37 °C, the SV40 T-antigen was inactivated for cell differentiation. Culture medium for human podocytes was RPMI 1640 Medium (Roth, Karlsruhe, Germany) with 10% fetal calf serum, 1% Penicillin/Streptomycin and 0.1% Insulin. We used the mirVana® miRNA mimic has-miR-26a-5p (miR-26a-5p mimic, Life Technologies, Carlsbad, CA, Catalog # 4464066) and *mir*Vana® miRNA mimic negative control #1 (miR-CTRL mimic, Life Technologies, Carlsbad, CA Catalog #4464058) for cell culture experiments in human podocytes. These miR mimic are small, chemically modified, double-stranded RNAs that mimic endogenous miRNAs and enable miRNA functional analysis by up-regulation of miRNA activity. MirVana™ miRNA mimics exhibit maximum and consistent effect *in vitro* at low concentration. They offer superior specificity due to unique Star strand modification. MirVana™ miRNA mimic negative control #1 is a random sequence miRNA mimic molecule that has been extensively tested in human cell lines and tissues and validated to not produce identifiable effects on known miRNA function. Seven days differentiated cultured human podocytes were transfected with 100 pM miR-26a-5p mimic/miR-CTRL for 4 h using Lipofectamin and Opti-MEM Medium (Thermo Fisher scientific, Waltham, MA) according to manufactures protocol. We performed a reverse transfection approach recommended by the company. Reverse transfection is faster to perform than forward transfection and is the method of choice for high-throughput transfection.

### Immunofluorescent staining of podocyte actin cytoskeleton

Cultured human podocytes were grown on cover slides and transfected with miR-26a-5p mimic or CTRL-mimic as described above. Three days after transfection slides were fixed at −20 °C for 10 min using ice-cold methanol and permeabelized using 0.1% Triton. After blocking with 10% donkey serum, immunofluorescent labeling of F-actin was done by incubation with Alexa Fluor® 546 phalloidin (Invitrogen) at 4 °C overnight. Finally, slides were mounted on glass slides using Vecta Shield with DAPI (Vector laboratories, Burlingame, CA, USA).

### qPCR in cultured human podocytes

For mRNA reverse transcription 1µg RNA, Oligo(dT)primer (Promega, Madison, WI, USA), and Random primer (Promega, Madison, WI, USA) were incubated at 70 °C for 10 min followed by an incubation with M-MLV RT buffer (Promega, Madison, WI, USA), dNTPs (Roche, Mannheim, Germany), and M-MLV reverse transcriptase (Promega, Madison, WI, USA) at 42 °C for 90 min and at 70 °C for 10 min.

Sybr green-based real-time PCR was performed with the following protocol: 1 minute at 95 °C followed by 35 cycles of 10 seconds at 95 °C, 10 seconds at 60 °C, and 10 seconds at 72 °C followed by 5 seconds at 95 °C and 1 minute at 65 °C. Individual samples were run in triplicate.

### *In vivo* studies in zebrafish

Research utilizing zebrafish reported in this publication was supported in part by Institutional Development Awards (IDeA) from the National Institute of General Medical Sciences of the National Institutes of Health under grant numbers P20GM0103423 and P20GM104318.

Zebrafish were grown and mated at 28.5 °C. Larvae were kept and handled in standard E3 solution as previously described^61^. Injection of vegf-Aa-MO (30 µM, 75 µM, 150 µM and 250 µM, vegf-Ab-MO (30 µM, 75 µM and 150 µM), CTRL-MO (150 µM) and Vegf-Aa 165 protein (2.5 µg/µl, 1247-ZV, R&D systems, Minneapolis, MN) in zebrafish eggs at one to two cell stage was done as previously described^25^.

The sequence of the morpholinos was 5′CATAGACTTTAACAGACATACCTGC3′ for vegf-Aa-MO, GCTGGAATGAGAATACTTACCGACA for vegf-Ab-MO and 5′CCTCTTACCTCAGTTACAATTTATA3′ for CTRL-MO.

Injection of miR mimics (mirVana® miRNA mimic has-miR-26a-5p (miR-26a-5p mimic, Life Technologies, Carlsbad, CA, Catalog # 4464066) and mirVana® miRNA mimic negative control #1 (miR-CTRL mimic, Life Technologies, Carlsbad, CA Catalog #4464058) in a concentration of 25 µM was done by microinjection in zebrafish eggs at one to two cell stages or in the cardinal vein of the fish at 48 hpf as previously described^25^.

### Eye assay in zebrafish

To confirm that the observed zebrafish phenotype was caused by damage of the renal filtration barrier we used a transgenic zebrafish that expresses a fluorescent Vitamin D binding protein Tg(l-fabp:DBP:eGFP fish). If plasma proteins are retained in the vascular system, the systemic fluorescence increases from over time and can easily be seen in the fish eyes. The maximum fluorescence intensities of grayscale images of the pupil of the fish were measured using Image J (Version 1.48 Wayne Rasband National Institutes of Health, USA) and reported in relative units of brightness (eye assay). A detailed description of the eye assay can be found in a publication of *Hanke et al*.^25,26^. The Mount Desert Island Biological Laboratory (MDIBL) animal care committee approved the animal protocol.

### Tail vessel pictures in of zebrafish

Pictures of the tail vessel were taken in Tg(l-fabp:DBP-eGFP/flk-mcherry) transgenic zebrafish that express with red fluorescent endothelium and a green fluorescent Vit. D binding plasma protein with the help of laser scanning confocal fluorescence microscopy at 96 hpf. Fish laying on their side were fixed in agarose

### Western blot

10 μg protein lysate of whole zebrafish (pooled from 12 animals per group) or 10 µg of podocyte cell lysate were resolved in 10 % SDS-PAGE and transferred to a polyvinylidene difluoride membrane. Detection of protein bands was performed using horseradish peroxidase-labelled secondary antibodies and visualized using enhanced chemi-luminescence reagents (Pierce, Rockford, IL). Primary antibodies were monoclonal mouse anti-zebrafish Vegf-A antibody (R&D systems, MAB1247, Minneapolis, MN 1:1000) and anti-GAPDH (Santa Cruz, FL-335, Dallas, Texas, 1:1000). Secondary antibodies were donkey anti-mouse IgG HRP (Santa Cruz, Dallas, Texas, 1:10.000) and donkey anti-rabbit IgG HRP (Santa Cruz, Dallas, Texas 1:10.000).

### Transmission electron microscopy of zebrafish glomeruli

Zebrafish larvae are fixed at 120 hpf in solution D and embedded in EPON (recipe/protocol from EMS, Hatfield, PA 19440, USA). We perform semi-thin (300 nm) and ultra-thin (90 nm) sectioning with a Leica UC-6 Microtome followed by transfer onto copper slit grids (EMS, Hatfield, PA 19440, USA). Staining of the grids was done with uranylacetat (2%) for 30 min and lead citrate for 15 min with three washing steps in between. Imaging was done with a JOEL JEM-1230 transmission electron microscopy.

### Ethics Statement

Animal zebrafish experiments were conducted according to the guidelines of the adherence to the NIH Guide for the Care and Use of Laboratory Animals and were approved by Institutional Animal Care and Use Committee of the Mount Desert Island Biological Laboratory, Maine (IACUC protocol#1406). All efforts were made to minimize the number of animals used and their suffering.

Ethical approval for the use of urines samples from patients with preeclampsia and controls was obtained from Ethics Committee of the Hanover Medical School (#1709–2013). All patients and controls gave informed consent. All research was performed in accordance with relevant regulations.

### Data analysis/statistics

We used the delta-delta cycle threshold (CT) method to normalize PCR data and to generate fold changes in miR expression. The fold change is the relative quantitative value calculated by 2^−(delta-delta CT)^. Delta-delta CT = delta CT (sample) − delta CT (reference) with delta CT (sample) = CT value for sample normalized to endogenous normalization gene and delta CT (reference) = CT value for calibrator compared to endogenous normalization genes. Endogenous normalisation was done with U6 snRNA for cells in miR TaqMan qPCRs, U6 snRNA and cel-miR-39 for urine in miR TaqMan qPCR and GAPDH in mRNA syber green PCRs.

All data are shown as means ± SD and were compared by ANOVA or students t-test (if only 2 groups) to look for statistical significance. Data were normally distributed.

## Electronic supplementary material

Supplementary Figure
