# Supplementary material for: Overexpression of preeclampsia induced microRNA-26a-5p leads to proteinuria in zebrafish
Source: Sci Rep. 2018 Feb 26;8:3621. doi: 10.1038/s41598-018-22070-w (PMC5827519; doi:10.1038/s41598-018-22070-w)

Overexpression of preeclampsia induced microRNA-26a-5p leads to proteinuria in zebrafish

Janina Müller-Deile[1,2], Patricia Schröder[2], Lynne Beverly-Staggs[2], Rebecca Hiss[1], Jan Fiedler[3], Jenny Nyström[4], Thomas Thum[3], Hermann Haller[1,2], Mario Schiffer[1,2]

[1]Department of Medicine/Nephrology, Hannover Medical School, Hannover, Germany

[2]Mount Desert Island Biological Laboratory, Salisbury Cove, Maine, USA

[3]Institute of Molecular and Translational Therapeutic Strategies, Hannover Medical School, Hannover, Germany

[4]Department of Physiology, Institute of Neuroscience and Physiology, the Sahlgrenska Academy, University of Gothenburg, Gothenburg, Sweden

Supplementary figure 1

miR-26a-5p does not bind to VEGF-A directly in cultured human podocytes.

A: Binding sites of miR-26a-5p seed region to human VEGF-A and zebrafish vegf-Aa. Binding position in the 3'UTR region is given next to the mRNA sequence.

B, C: Luciferase assay showing that there is neither a direct interaction between miR-26a-5p and VEGF-A in a miR-26a-5p mimic concentration of 50 nM (B) nor in a miR-26a-5p mimic concentration of 100 nM (C).

D: Luciferase reporter assay to validate miR-26a-5p binding to PIK3C2α was performed in human embryonic kidney (HEK) cells 293. Cells were lysed 24 hours after transfection and subsequently used for luciferase activity. Luciferase reads were normalized with b-galactosidase values. Concentration of the miR-26a-5p mimic was 50 nM. *$P < 0.05$.

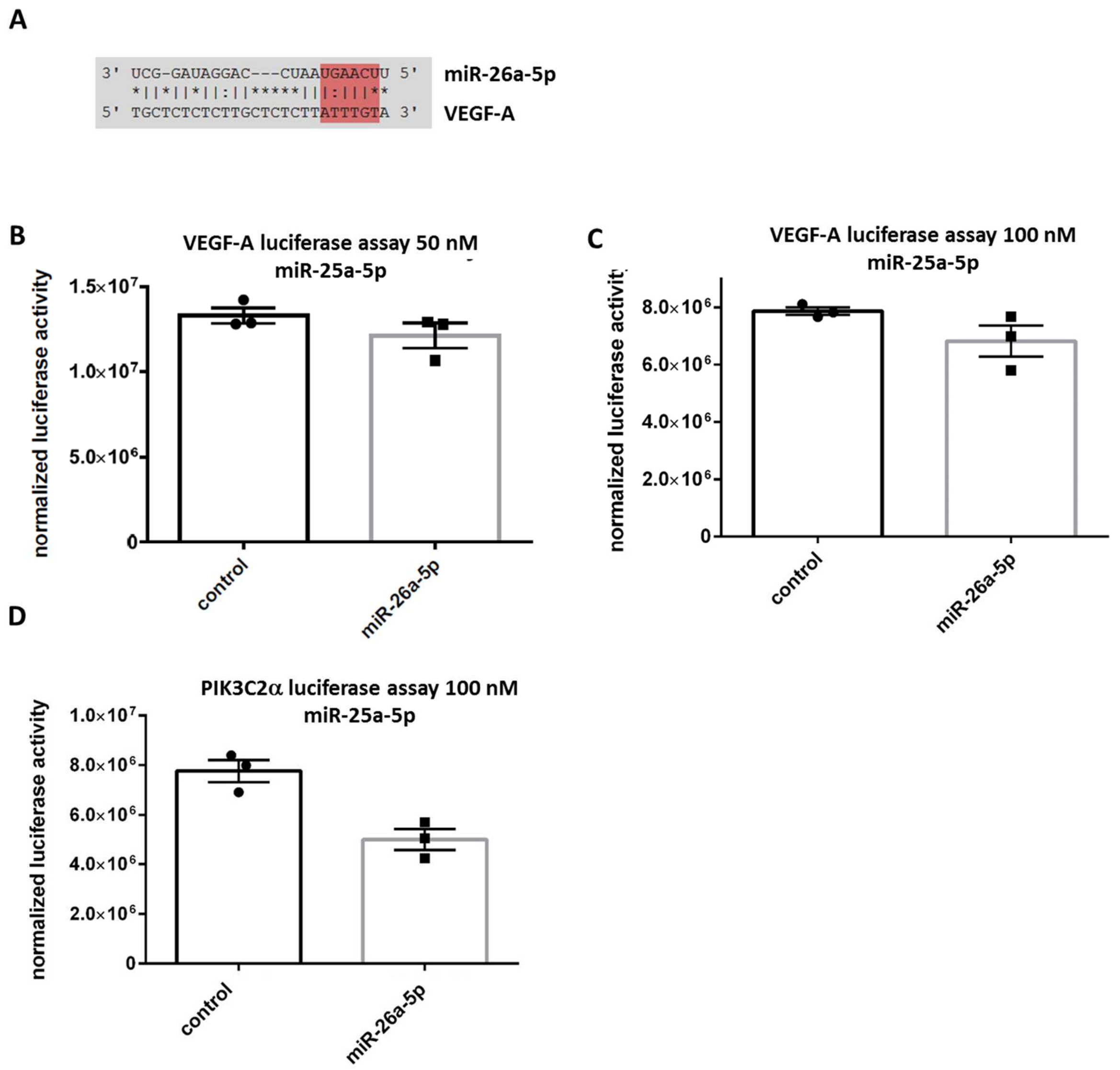

Supplementary figure 2

Three independent western blots from the statistic shown in *Fig. 1 E*

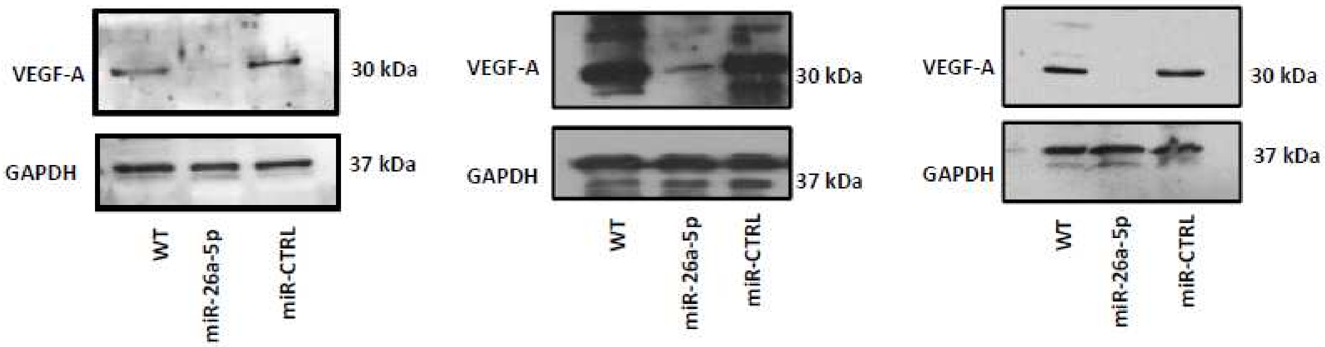

Supplementary figure 3

Full-length blots of *Fig. 4A.*

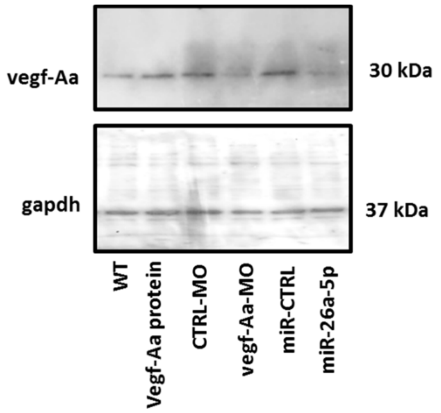

Supplement: Supplementary file 1 — Supplementary Figure [file 41598_2018_22070_MOESM1_ESM.pdf]
